# Blood derived extracellular vesicles in patients with glioblastoma: preliminary experience from a monoinstitutional series

**DOI:** 10.1007/s12094-026-04223-w

**Published:** 2026-02-04

**Authors:** Andrea Di Cristofori, Martina Ghizzi, Francesca Raimondo, Alberto Ramponi, Andrea Cimino, Martina Giambra, Francesca Graziano, Gianpaolo Basso, Marialuisa Lavitrano, Giorgio Carrabba, Carlo Giussani, Angela Bentivegna

**Affiliations:** 1 Neurosurgery, Fondazione IRCCS San Gerardo Dei Tintori, 20900 Monza, MB Italy; 2 School of Medicine and Surgery, University of Milano-Bicocca, 20900 Monza, Italy; 3 GBM-BI-TRACE (GlioBlastoMa-BIcocca-TRAnslational-CEnter), University of Milano-Bicocca, 20900 Monza, Italy; 4 PhD Program in Neuroscience, University of Milano-Bicocca, 20900 Monza, Italy; 5 Biostatistics and Clinical Epidemiology, Fondazione IRCCS San Gerardo Dei Tintori, Monza, Italy; 6 Bicocca Center of Bioinformatics, Biostatistics and Bioimaging (B4 Centre), School of Medicine and Surgery, University of Milano-Bicocca, Monza, Italy; 7 Neuroradiology Unit, Fondazione IRCCS San Gerardo Dei Tintori 20900, Monza, MB Italy; 8 UC Medical Genetics, Fondazione IRCCS San Gerardo Dei Tintori, 20900 Monza, MB Italy

**Keywords:** Glioblastoma, Extracellular vesicles, Liquid biopsy, Chemotherapy, Radiotherapy, Neuro-oncology

## Abstract

**Purpose:**

Glioblastoma (GB) is the most common and aggressive primary brain tumor in adults, with its significant inter- and intra-tumoral heterogeneity being a major factor in its treatment resistance and overall prognosis. GB diagnosis typically involves magnetic resonance imaging, confirmed by histology after surgical resection or biopsy. Recurrence is almost expected despite adjuvant therapies. Extracellular vesicles (EVs) may represent promising cancer biomarkers for diagnosis, prognosis, and therapeutic monitoring.

**Methods:**

In this work, we monitored 21 GB patients at different time intervals performing a quantitative and dimensional analysis of plasma-derived EVs, with the aim of finding correlations with their clinical course.

**Results:**

Our analyses revealed a slight correlation with patients’ clinical conditions during follow-up, such as tumor time recurrence over time, but no significant difference in plasma EV concentration in GB patients and healthy control subjects (HC), contrary to previously published data.

**Conclusions:**

Although based on a limited number of patients, our methodological study highlights the need for a universal analysis method to compare data from large patient populations in order to use EVs as a biomarker for the diagnosis of recurrence by liquid biopsy, especially in GB, a tumor known for its heterogeneity.

**Supplementary Information:**

The online version contains supplementary material available at 10.1007/s12094-026-04223-w.

## Introduction

Glioblastoma (GB) is the main primary malignant tumor of the brain. Despite efforts in research, it is still without a cure and treatments encompass maximal surgical resection followed by adjuvant therapies (mainly chemo- and radiation therapy) [1, 2].

GB management is based on brain MRIs that are mandatory to plan patient treatments and follow-up. In some situations, the adjunct techniques such as perfusion scans, spectroscopy, or PET imaging are used to distinguish between tumor recurrence, pseudoprogression or radionecrosis; however, in several cases all imaging options are inconclusive [3]. An accurate diagnosis of GB recurrence has therapeutic and prognostic implications, including the need for a second surgery or a change in the kind of treatments [4, 5]. For such reasons, the clinicians ask for a blood marker for diagnosis and follow-up [6]. Liquid biopsy has emerged in recent years as a promising advancement for early diagnosis, monitoring, and prognosis of GB patients [7, 8].

The extracellular vesicles (EVs) are nanosized membranous particles released into biological fluids by cells in physiological and pathological conditions. Their main function is intercellular communication transporting specific molecular cargoes to target cells. Since EVs reflect the molecular composition of the cells of origin, they are considered a potential source of biomarkers for liquid biopsy [9].

Some studies highlight that the concentration of EVs increases quantitatively in patients diagnosed with GB compared to healthy control subjects (HC), since they are involved in tumor initiation and progression [10]. According to several authors, EV concentration decreases after surgical resection, radiotherapy, or chemotherapy, and rises again in case of tumor recurrence, making these structures potential biomarkers for diagnosis, monitoring and prognosis of GB patients [3, 11, 12]. Despite these interesting findings, there is no clear standardized method to isolate and count EVs, and no official guidelines currently exist [13, 14].

Starting from these premises, we monitored 21 patients diagnosed with GB at different time points, performing a quantitative and dimensional analysis of plasma-derived EVs with the aim of finding correlations with their clinical course. The final purpose was to propose an alternative diagnostic and prognostic approach to those currently in use.

## Materials and methods

### Study population and blood sampling

A total of 21 adult patients were consecutively enrolled in the study from January 2020 to January 2024. Histological diagnosis was assessed or reviewed according to the 2021 WHO classification of Central Nervous System tumors [15]. Patients underwent surgical resection at Fondazione IRCCS Ospedale San Gerardo dei Tintori in Monza, Italy. Extent of surgical resection (EOR) was evaluated with an early post-operative MRI as described by Berger [16] using BrainLab™ segmentation software (BrainLab™, Germany). Fluid attenuated inversion recovery (FLAIR) hyperintense peritumoral region was also segmented. After surgery, the patients were treated with radiotherapy (RT) and chemotherapy (temozolomide, TMZ) according to Stupp’s protocol [17]. Disease monitoring involved an MRI scan and a neuro-oncological visit every 3 months. Follow-up with blood sampling ended 9 months after surgery. A total of 103 samples were analyzed (Supplementary materials, Table S1). Five peripheral blood samples were collected from each patient: before and after surgical resection (Basal and Post-OP, respectively), and during each follow-up control (FU-1, 3 months after surgery; FU-2, 6 months after surgery; and FU-3, 9 months after surgery).

**Fig. 1:**
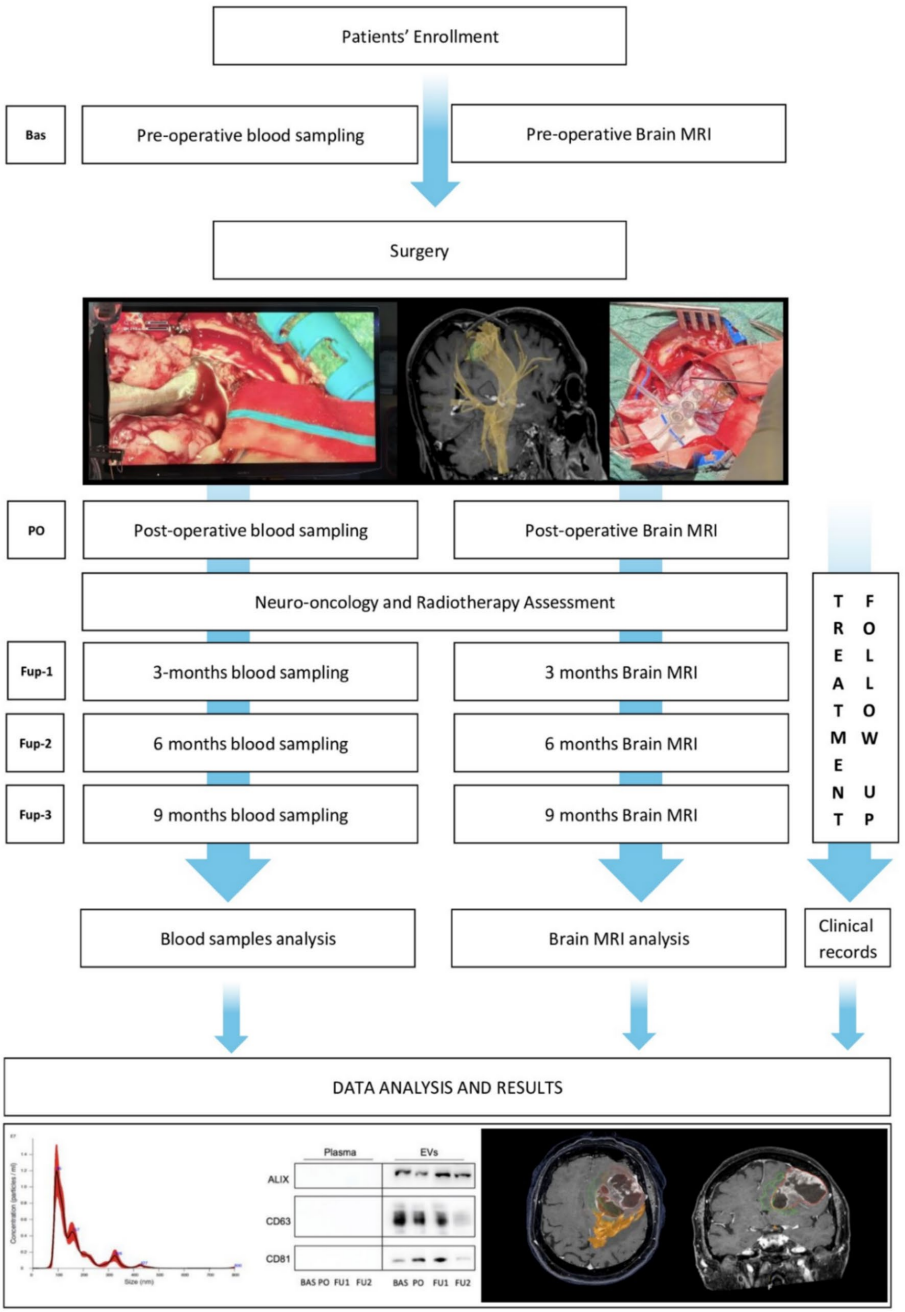
Flowchart of the experimental design

Tumor recurrence was diagnosed according to the RANO criteria [18]. The peripheral blood samples were also collected from eight HCs for comparison with GB patients. A chronological workflow diagram is reported in Fig. 1.

### Patient stratification according to clinical findings

Clinical subdivision based on patients’ clinical findings was used to determine whether EVs could be a suitable and reliable biological marker for translation into clinical practice.

Progression free survival (PFS)

Patients were subdivided into two groups according to the PFS. Since in our historical cohort of patients and in large case series median PFS is 9 months, we used this value as a cut off [19, 20]. Patients that experienced a tumor recurrence within 9 months were allocated to the “early relapse” group, while the others to the “late relapse” group.

Radiological* correlation*

**Fig. 2:**
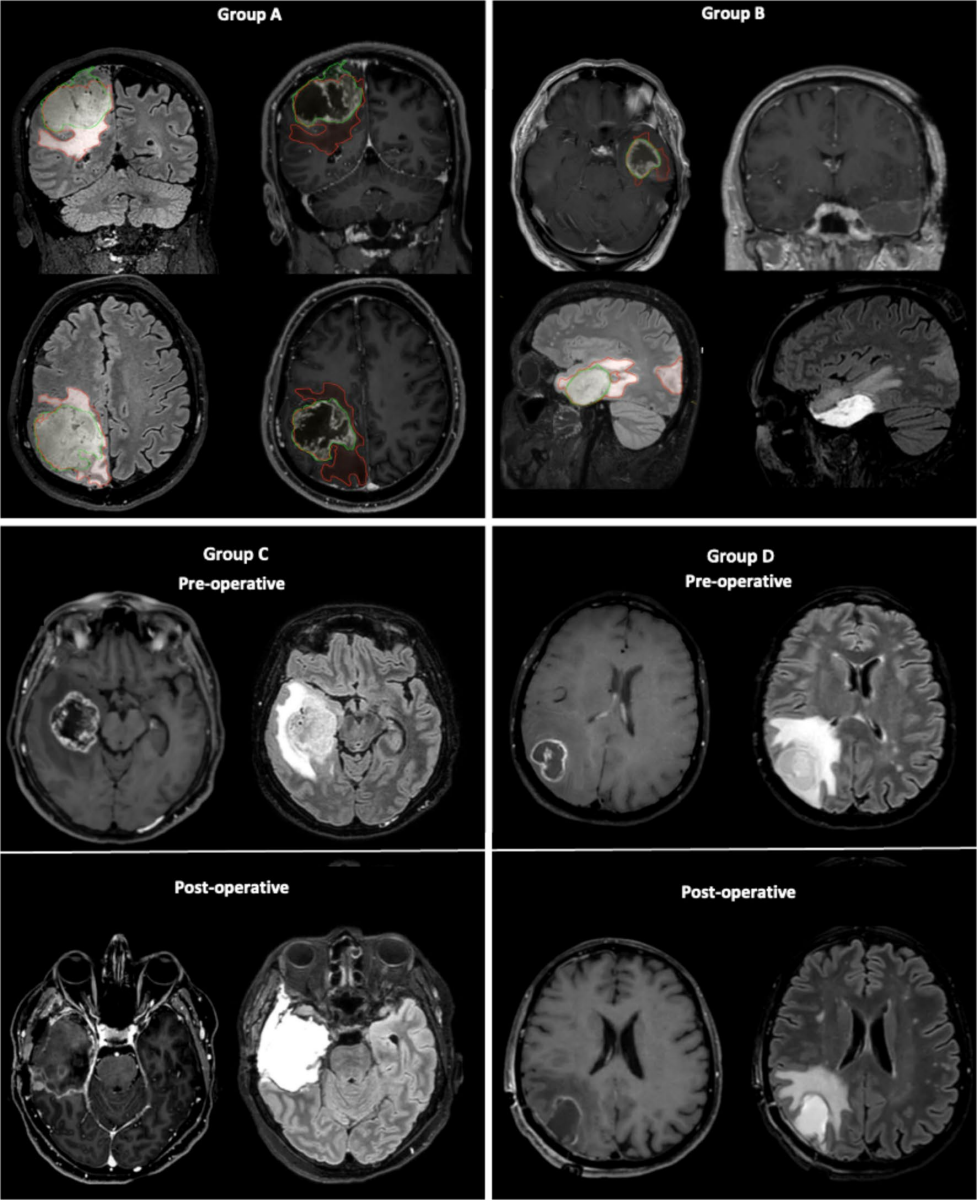
Some examples of patient stratification based on neuroradiological features. Top row, left panel: case of a patient included in group A affected by GB presenting a single lesion; top row, right panel: case of a patient included in group B presenting a multifocal lesion. Areas segmented in green: T1 weighted contrast enhancing part of the tumor. Areas segmented in red: tumoral FLAIR hyperintensities. Bottom row, left panel: Group C patient who received GTR plus complete resection of perilesional FLAIR hyperintensities; bottom row, right panel: Group D patient who received contrast-enhanced tumor GTR without removal of perilesional FLAIR hyperintensities

Based on neuroradiological features, considering both contrast-enhancing tumor and FLAIR hyperintensities, two patient groups were identified: patients with a single contrast-enhancing lesion and perilesional hyperintensities (group A), and patients with multifocal lesions (group B). We chose to study peritumoral FLAIR hyperintensities because they may represent perilesional tumor infiltration or may represent distant localizations of gliomas in the case of multifocal tumors. Moreover, such hyperintensities may play an active biological role and may be involved in EV release in the bloodstream (Fig. 2, top panel shows two representative cases).

*Extent of *surgical* resection (EOR)*

We also analyzed the relation between EVs and the surgical resection. Group C included patients with gross total resection (GTR) and complete resection of FLAIR hyperintensity; while group D consists of patients with GTR resection and residual FLAIR hyperintensity (Fig. 2, bottom panel shows two representative cases). Patients with sub-total resections (STR) were excluded from this analysis.

### Sample processing

Two Vacutainer tubes with sodium citrate were collected and processed within 2 h. The samples were centrifuged at 2400 xg for 15 min at 4 °C to obtain plasma samples, and then at 3000 xg for 10 min at 4 °C discarding about 250 µl from the tube bottom, to obtain platelet-free plasma (PFP); aliquots were stored at −80 °C.

### Extracellular vesicle isolation

Plasma EVs were isolated by differential centrifugation and ultracentrifugation [13, 14]. Briefly, PFP (1–2 ml) was thawed and diluted with an equal volume of PBS and centrifuged at 20 °C at 2000 xg for 30 min. The supernatants were further diluted by adding 4 volumes of PBS and centrifuged at 12,000 xg for 45 min. The supernatant was filtered through a 0.22-μm syringe filter, then ultracentrifuged at 200,000 xg for 120 min. The pellets containing crude EV fractions were suspended in PBS and washed by ultracentrifugation in the same conditions. EV pellets were dissolved in sterile PBS, and stored at −80 °C.

### Nanoparticle tracking analysis of extracellular vesicles

Size and concentration of EVs were measured by nanoparticle tracking analysis (NTA) using NanoSight NS300 (Malvern Preanalytical Ltd, Malvern, UK), equipped with a 488-nm laser (blue), a sCMOS camera, and a syringe pump. EVs were diluted 1:50 in sterile PBS and injected into the microfluidic chamber using a syringe pump with infusion rate of 30 AU at 25 °C. The capture was performed through three videos of 60 s to obtain 1500 frames for measurement. Tracking of each nanoparticle was recorded and the size distribution profiles and EV concentration analysis were performed through NTA Software (3.3 version).

### Western blotting (WB)

WB and immunodecoration were performed to evaluate the enrichment of typical EV markers (Alix, CD63, and CD81). Equal amounts of proteins were separated into 4–12% Bolt Bis–Tris precast polyacrylamide gels using MOPS SDS running buffer (Life Technologies) and transferred onto nitrocellulose filter. Blocking, antibody incubations and washings were performed in blocking solution (5% free-fat milk, 0.2% Tween-20 in PBS). The primary and secondary antibodies were used according to manufacturer dilutions: rabbit pAb anti-Alix 1:1.000, mouse mAb anti-CD63 1:250 and anti-CD81 1:500, and anti-rabbit and anti-mouse IgG secondary antibodies conjugated with horse-radish peroxidase 1:10.000 were from ThermoFisher Scientific™ (Massachusetts, USA). Protein signals were developed using the Enhanced Chemiluminescence Assay (ECL) (SuperSignal Western Blot substrates, ThermoFisher). Images were acquired by the CCD Camera Amersham Imager 600 (Cytiva), and densitometric analysis of protein bands was performed by ImageQuant TL Software 8.1 (GE Healthcare).

### Statistical analysis

Descriptive characteristics were reported as frequency and percentage for categorical data, mean and standard deviation (SD) or median, and I-III quartile for continuous variables, where appropriate. Comparison between baseline characteristics was performed through the Mann–Whitney U test, or t-test depending on the nature of the variable (median or mean values).

Paired t-test and repeated measures ANOVA were performed to compare the values across the different follow-up times. A two-tailed *p* value of 0.05 was considered significant. Since some analyses involved multiple tests, the p-values were adjusted with a Bonferroni correction.

### Ethics

This study was approved by the ethics committee “Comitato Etico Monza e Brianza” (study number: 0031436-GLIODRUG-V, January 2020). Written informed consent was obtained from patients.

## Results

### Clinical data

A total of 21 consecutive patients were operated on for a GB. The patients’ clinical features are reported in Table 1. Radiologically, there were 16 single lesions and 5 multifocal tumors. All patients were affected by an IDH wild-type GB and presented homogeneous features. No significant post-operative complications after surgery were reported. Six patients received an STR, while 15 received a GTR of contrast-enhancing tumor. Among patients who received GTR, 9 received a complete removal of the FLAIR perilesional hyperintensity. Survival analysis according to EOR and MGMT methylation is reported in Supplementary material, Figure. S1.

**Table 1:** Clinical, radiological and histological data of GB patients

	Clinical characteristics											First line therapy		
	Sex	Age	Location	Lesion	PFS	OS	GTV CE	GTV FLAIR	MGMT	EOR	Death yes/no	Radio	Chemo	Second line therapy
PZG7	M	55	Right parietal lobe	Single	5	15	9.05	121.05	No	GTR	Yes	RT	TMZ	Fotemus
PZG8	M	58	Right insular temporo lobe	Multifocal	17	33	66.2	49.6	Yes	GTR	Yes	RT	TMZ	Fotemus
PZG13	M	59	Left temporal lobe	Multifocal	9	14	3.79	83.31	Yes	GTR	Yes	RT	TMZ	Fotemus
PZG15	M	79	Left frontal lobe	Single	10	16	43.3	missing	No	GTR	Yes	RT	TMZ	Fotemus
PZG17	M	82	Right frontal lobe	Single	4	13	13.5	missing	No	GTR	Yes	RT	TMZ	–
PZG19	M	61	Right parieto occcipital lobe	Single	10	16	89.3	140.9	Yes	GTR	Yes	RT	TMZ	Fotemus
PZG20	F	60	Left occipital lobe	Single	34	46	40.4	79.5	No	GTR	No	RT	TMZ	Regorafenib
PZG22	M	59	Left temporal lobe	Multifocal	19	40	25.2	45.1	Yes	GTR	Yes	RT	TMZ	Fotemus
PZG25	F	78	Right temporo occipital lobe	Single	6	8	4.81	2.66	Yes	GTR	Yes	RT	TMZ	Fotemus
PZG26	F	70	Left temporal lobe	Single	8	30	45.8	7.2	Yes	STR	No	RT	TMZ	–
PZG31	M	68	Left parietal lobe	Single	8	10	22.6	10.9	No	GTR	Yes	RT	TMZ	Regorafenib
PZG33	M	55	Right occipital lobe	Single	1	32	30.1	103.6	Yes	GTR	Yes	RT	TMZ	Regorafenib
PZG35	F	55	Right occipital lobe	Single	33	36	72.9	23.7	Yes	GTR	Yes	RT	TMZ	Regorafenib
PZG43	M	54	Right parietal lobe	Single	4	20	86.9	63.9	Yes	STR	Yes	RT	TMZ	Regorafenib
PZG46	M	62	Right frontal temporo insular lobe	Single	16	16	24.8	2.7	No	STR	Yes	RT	TMZ	Regorafenib
PZG55	M	57	Left temporal lobe	Multifocal	4	16	6.84	12.9	Yes	GTR	Yes	RT	TMZ	Regorafenib
PZG62	M	62	Left occipital lobe	Single	11	12	42.1	14.9	Yes	STR	Yes	RT	TMZ	Regorafenib
PZG64	M	50	Left parietal lobe	Multifocal	17	17	19.7	83.1	No	STR	No	RT	TMZ	–
PZG67	F	74	Right frontal lobe	Single	8	14	34.5	66.3	Yes	GTR	No	RT	TMZ	Regorafenib
PZG71	F	73	Right temporal lobe	Single	14	14	16.6	127.8	Yes	GTR	Yes	RT	TMZ	–
PZG72	M	68	Right temporo parietal lobe	Single	6	15	51.6	61	No	STR	No	RT	TMZ	Regorafenib

*PZG* patient, *M* males, *F* females, *PFS* progression free survival, *OS* overall survival. PFS and OS are expressed in months after surgery. GTV CE indicates contrast enhancing gross tumor volume; GTV FLAIR indicates gross tumor volume of perilesional FLAIR positive hyperintensities. *MGMT* O-6-methylguanine-DNA methyltransferase: Yes, indicates promoter hypermethylation, no, indicates no promoter hypermethylation. *EOR* extent of resection, *GTR* gross total resection, *STR* subtotal resection, *RT* radiation-therapy, *TMZ* temozolomide, *Fotemus* fotemustine

### Extracellular vesicle analysis

NTA and EV marker enrichment (ALIX, CD81 and CD63) confirmed the quality of EV fractions (Supplementary material, Figure S2). In particular, NTA shows typical EV size distribution with a main peak of diameter around 100–150 nm.

**Fig. 3:**
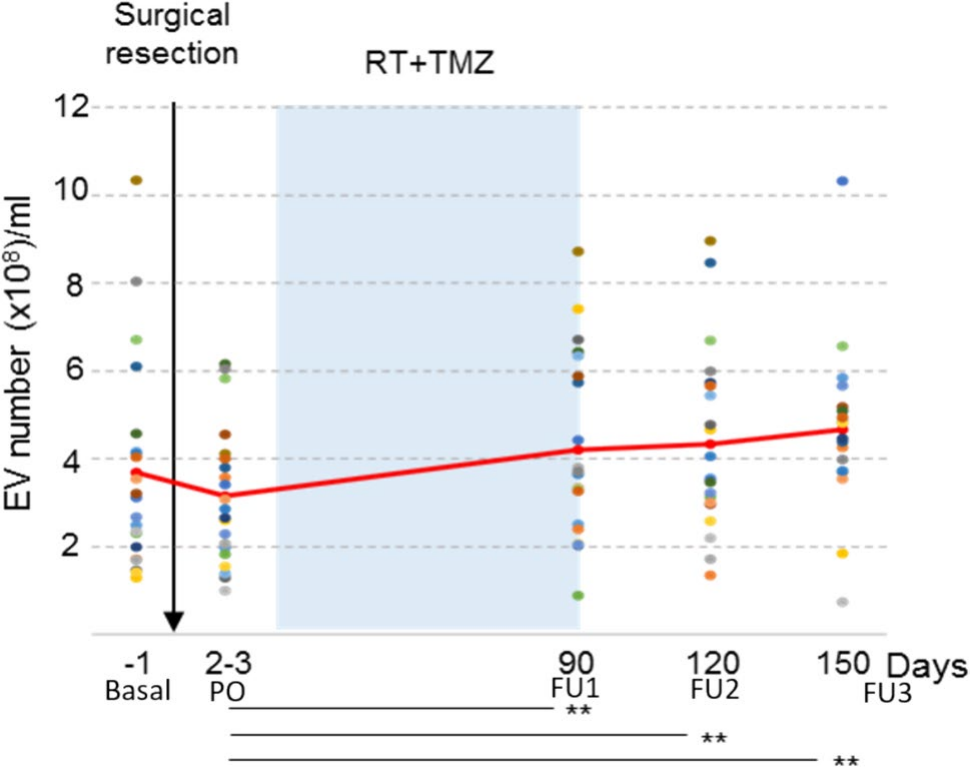
Longitudinal trend of plasma EV concentration for all patient enrolled. The red line shows the mean EV concentration (particle/plasma ml) of all patients. Each colored dot represents the value of a single patient. The black arrow represents the time of surgical resection; day −1, plasma collection before surgery (basal); day 2–3, after surgery (PO); day 90, follow-up 1 (FU1: 76–105 days, 3 months); day 120, follow-up 2 (FU2: 109–130 days, 6 months); day 150, follow-up 3 (FU3: 140–165 days, 9 months); RT + TMZ, Radiotherapy plus temozolomide. Mann Whitney test between post-surgery and follow-up: **p value < 0.01, *p value < 0.05

Figure 3 shows the global overview of the number of EVs overtime during the neuro-oncological follow-up of patients, independently of their radiological or surgical status. Overall, the patients in our cohort showed a decrease of EV concentration after surgery (not significant) and a progressive increase after concomitant chemo-RT (FU-1). Individual patient analyses are reported in the Supplementary material, Figure S3.

### Correlation of EV concentration with clinical features

**Fig. 4:**
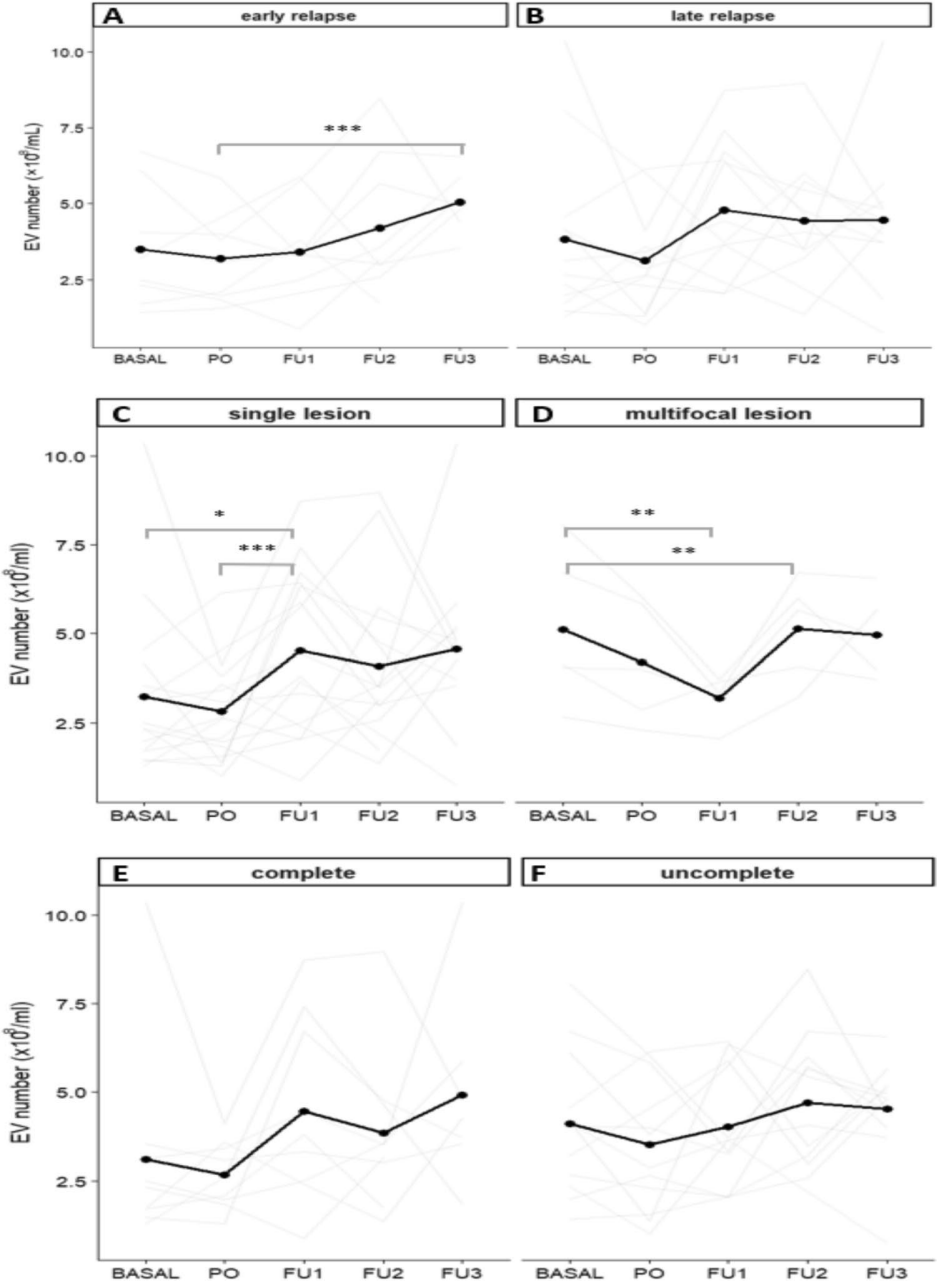
Longitudinal trend of EV concentration during the neuro-oncological follow up according to clinical stratification. Panel A and B—Time of recurrence: **A** early relapse; **B** late relapse. Panel C and D – Lesion type: **C** single lesion tumor; **D** multifocal tumor. Panel E and F—type of surgical resection: **E** complete surgical resection of FLAIR hyperintense peritumoral zone; **F** uncomplete surgical resection of FLAIR hyperintense peritumoral zone. Gray lines represent individual patient trajectories; black lines indicate group means; asterisks refer to the significant differences between timepoints in the subgroups (***p < 0.01, **p < 0.05, and *p = 0.05). *BAS* preoperative, *PO* postoperative, *FU1* follow up 1, *FU2* follow up 2, *FU3* follow up 3

According to the PFS, patients with early recurrence had a progressive increase of the number of EVs during the neuro-oncological follow-up (*p* < 0.01 comparing FU-3 to post-OP samples), while patients with late recurrence showed stable EV concentration (Fig. 4A and B).

Stratifying patients based on lesion type, single or multifocal, it was possible to appreciate a different trend in EV concentration. In fact, in patients harboring a single lesion, there was an increase of the mean EV number at the end of concomitant RT plus TMZ (after 3 months from surgery) (*p* < 0.01 for FU-1 *versus* post-OP samples, and *p* < 0.05 for FU-1 *versus* basal samples); while in patients presenting a multifocal lesion, there was a continuous decrease of EVs till the FU-1 checkpoint (*p* < 0.05 for FU-1 *versus* basal samples), then an increment of EVs at 6 months after surgery (*p* < 0.05 for FU-2 *versus* basal samples) (Fig. 4C and D). Moreover, considering only patients with a single lesion, we assessed no differences associated with the kind of surgical resection obtained. Patients in the single lesion group that had a GTR, with or without residual perilesional FLAIR hyperintensity, had substantially the same trend in variation of EV over time and showed an increase of EVs after RT (Fig. 4E and F).

**Fig. 5:**
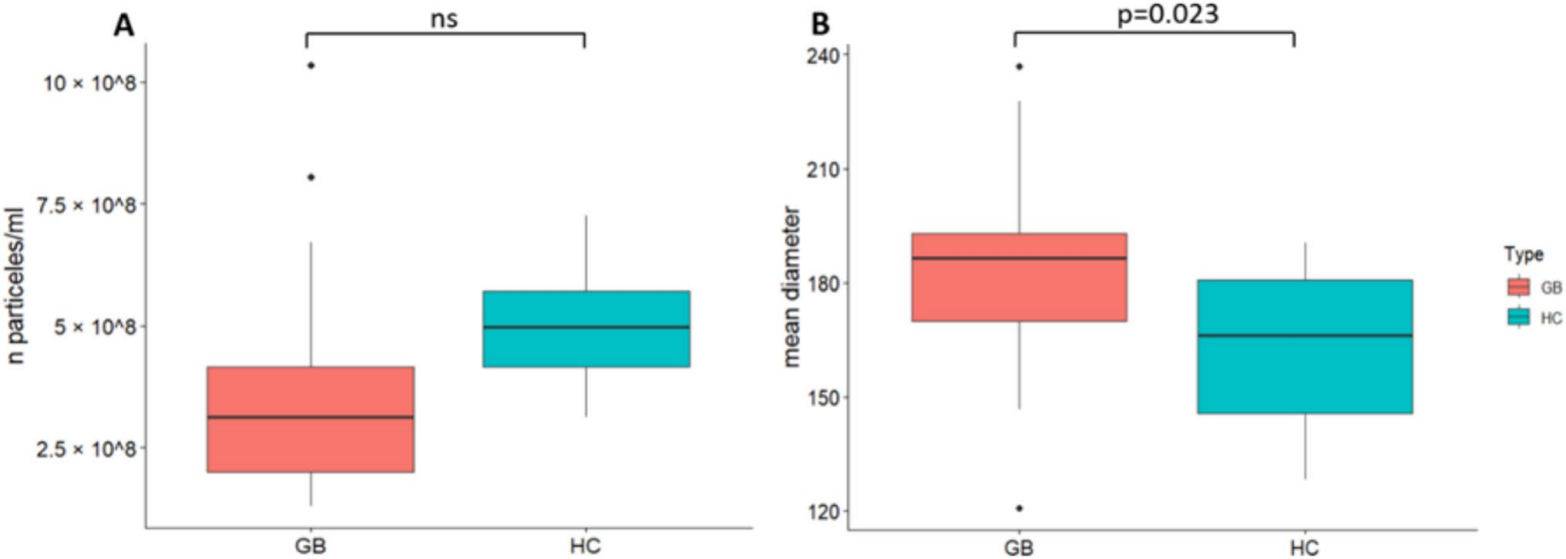
Comparison of plasma EVs from GB patients and healthy control subjects (HC). **A** Plasma EVs concentration (n particles/mL) in glioblastoma patients (GB) and healthy controls (HC). **B** Plasma EV mean diameter (nm) of GB patients and healthy controls (HC). The p-value refers to the t-test comparisons between the two groups; ns indicates no significant difference in EV concentration, while a significant difference was found in EV mean diameter

Finally, we evaluated plasma EVs from eight HCs. No difference in number of particles could be found in GB patients and HCs (Fig. 5A). Conversely, a significant increase in mean particle diameter is observed in GB patients compared to HCs (*p* = 0.023) (Fig. 5B).

## Discussion

Our Institution is one of the regional hubs for neuroscience in Northern Italy. Patients with GB are treated through a multidisciplinary approach within a well-defined neuro-oncological care pathway. Our work aimed to translate current advanced knowledge in neuro-oncology regarding liquid biopsy into our standard of care, with the goal to improve patient management and treatment. We structured a longitudinal follow-up with EV isolation concomitantly with clinical neuro-oncological follow-up to build up a translatable model from the laboratory to the outpatient clinic. Only a few reports are available about the use of EVs as GB biomarkers in patients during their neuro-oncological follow-up [10, 21]. These studies show that the plasma EV concentration is higher in GB patients compared with HCs. Moreover, in both works, concentration of plasma EVs is related to the presence of the tumor bulk, since tumor removal is associated with a drop in EV concentration, starting from post-operative day 1 [10, 21].

On the contrary, we found no significant difference in the plasma EV concentration of GB patients and HCs, but a mild difference in EV size. We also observed a slight decreasing trend in post-operative samples but it was not statistically significant. In our opinion, these results might be attributed to pre-analytical variables, from blood collection to the EV isolation protocol. We applied the available guidelines for EV isolation reported by the International Society for Extracellular Vesicles (ISEV) [13, 14], which specify several variables and methods for EV isolation and counting, without complete standardization. However, the lack of a clear straightforward method for isolating and counting EVs makes it difficult to compare one study with another.

We might hypothesize that there may be a number of causes for the discrepancy between our findings and those of previously published studies. Such considerations have been drawn up in two ways: comparing our isolation protocol with those reported in literature and summarizing the vast literature about blood EV isolation (Supplementary Table 2).

One of the main factors that we found is that cited works employed EDTA as anticoagulant [10, 21], whereas we chose sodium citrate. Our choice might have had a significant impact on our results. Specifically, Del Bene et al. found that EDTA is much better at stopping platelet activation than sodium citrate [10, 11] since platelet activation/aggregation causes EV release. On the contrary, Yang et al. suggested that sodium citrate maintains better physical properties and surface marker protein expression of plasma EV [22]. In ISEV guidelines of 2018, it was reported that neither EDTA nor sodium citrate should be used as anticoagulants; although other societies (e.g., the Scientific Standardization Committee of the International Society on Thrombosis and Haemostasis) recommended the trisodium salt of citrate [14, 23].

Second, some researchers recommended avoiding butterfly cannulas, commonly used in clinical practice, in favor of straight needles. We decided to standardize our way of collecting biological samples and to adhere to standard medical practices in use at our neuro-oncology department; but this choice may be a confounding factor that can be found in other studies as well [24].

As mentioned above, in contrast to the results published by others [10], we did not find a significant decrease in EV plasma concentration in our patients following surgery. This discrepancy could be explained by the inflammatory state caused by the surgical removal of the tumor, which might influence vesicles release [25]. Although not demonstrated, we thought the lower vesiclemia found in GB patients compared to HCs might be related to the steroid administration. In fact, nearly all of our patients received high doses of dexamethasone before to and immediately following surgery. Indeed, the bone marrow, which produces most of the EVs in the blood, may be impaired in its ability to function while patients are taking corticosteroids.

Despite these concerns, our data may suggest a correlation between plasma EV concentration and the clinical conditions of patients during follow-up. Interestingly, we observed different trends in plasma EV levels according to the time of recurrence and with the kind of radiological presentation.

Generally, after concomitant TMZ and RT, there is a reduction of circulating EVs until tumor relapse [10, 21, 26, 27]; although some studies did not report a checkpoint measure but only the EVs count when the tumor recurs [10]. On the other hand, previous results contradict what is found from in vitro studies on malignant tumors. In particular, Arscott et al. [28] described that GB stem cells release EVs when irradiated in vitro. Our study would confirm the same behavior in vivo in GB patients, suggesting the presence of a residual cellular population (cancer stem cells?) that releases EVs after RT. Such a population would be responsible for a subsequent recurrence after adjuvant treatments with a more aggressive behavior, especially when RT and chemotherapy do not suppress the production of EVs (as has been found in early recurrent tumors—Fig. 4). Multifocal tumors showed a delayed EVs release (FU-2), maybe suggesting that the adjuvant therapies suppress the production of both contrast-enhancing and non-enhancing tumors. On the other hand, healthy brain tissues may respond to RT by increasing EVs. Unfortunately, we were not able to study the content of EVs. Probably, such an approach could lead to a better understand of the pathways involved in EV release in response to adjuvant chemo-RT. Interestingly, all patients distinguished by radiological presentation (in a tailored view) showed an increase of EVs after chemo-RT. If confirmed on a broader cohort of patients, we believe that assessment of plasma EV could support patient monitoring and predict relapse.

## Limitations

Our work aims to investigate the possible role of EVs as a biological marker for GB. Our results are preliminary, and they are influenced by several limitations, such as a small sample size, lack of a clear standardized protocol for EV isolation, and a small sample of healthy controls.

## Conclusion

Several pieces of biological evidence suggest that research on EVs is a landmark for oncology and precision medicine. However, standardization is crucial and our preliminary findings suggest the urgent need for improved methodological rigor. Until now, to the best of our knowledge, current guidelines are poor and the choice of EV isolation technique still largely depends on the type of analytical application [13, 29]. Improved methodological rigor through the development of an internationally accepted protocol will strengthen the reliability and reproducibility of EV studies conducted on large case series of patients.

If our preliminary findings were to be confirmed in further studies, it would have a high impact on future research pipelines. In particular, our findings would help to potentially redirect EV studies not on “vesiclemia” but on analysis of EV cargoes.

## Supplementary Information

Below is the link to the electronic supplementary material.

Supplementary file1 (DOCX 269 KB)

## Data Availability

The data used to support the findings of this study are available from the corresponding author upon request.
