# Supplementary material for: Blood derived extracellular vesicles in patients with glioblastoma: preliminary experience from a monoinstitutional series
Source: Clin Transl Oncol. 2026 Feb 4;28(8):3434–44. doi: 10.1007/s12094-026-04223-w (PMC13401546; doi:10.1007/s12094-026-04223-w)
Supplement: Supplementary file 1 — Supplementary file1 (DOCX 269 KB) [file 12094_2026_4223_MOESM1_ESM.docx]

**Supplementary Material**

Table S1: Timing of peripheral blood sampling for each patient in the analyzed cohort.

| **Timing of peripheral blood sampling** | | | | |
| --- | --- | --- | --- | --- |
| Patient | Postoperative (days) | Follow up 1 (months) | Follow up 2 (months) | Follow up 3 (months) |
| PZG7 | 2 | 3 | 6 | 10 |
| PZG8 | 2 | 3 | 5 | 8 |
| PZG13 | 4 | 4 | 7 | 10 |
| PZG15 | 4 | 3 | 5 | 9 |
| PZG17 | 4 | 3 | 6 | / |
| PZG19 | 9 | 3 | 6 | 9 |
| PZG20 | 2 | 3 | 5 | 8 |
| PZG22 | 4 | 2 | 5 | 9 |
| PZG25 | 4 | 2 | 6 | / |
| PZG26 | 4 | 3 | 6 | 9 |
| PZG31 | 2 | 2 | 5 | 8 |
| PZG33 | 4 | 2 | 6 | 8 |
| PZG35 | 2 | 3 | 6 | 10 |
| PZG43 | 2 | 2 | 6 | 9 |
| PZG46 | 4 | 2 | 6 | 9 |
| PZG55 | 2 | 2 | 6 | 9 |
| PZG62 | 3 | 4 | 7 | 10 |
| PZG64 | 3 | 2 | 4 | 8 |
| PZG67 | 4 | 2 | 6 | 9 |
| PZG71 | 2 | 3 | 5 | 8 |
| PZG72 | 1 | 3 | 5 | 8 |

Preoperative blood samples are all taken one day before surgery and are not reported in the table. Days indicate the number of days after surgery, months indicate the months after surgery. “PZG” indicates patient.

Table S2 Comparison of our study with the literature on the use of EVs as blood biomarkers in GB

| **Reference** | **Patient Sample Size** | **HCs** | **Anticoagulant Used** | **Timing of Blood Samples Collection** | **Methods of**  **EV Isolation** | **Methods of**  **EV Analysis** | **EV Mean Size:**  **GB *vs* HCs** | **Limitations** |
| --- | --- | --- | --- | --- | --- | --- | --- | --- |
| Ricklefs FL et al, Neuro Oncol. 2024  [21] | n=101 GB plasma samples pre-OP;  n=34 GB plasma samples 1 day post-OP;  n=40 GB plasma samples 4–6 days  post-OP | n=29 | EDTA | Pre-OP;  1 day post-OP;  4–6 days post-OP | Differential  centrifugation | NTA (LM14/638 nm laser)  TEM  IFCM | No difference | - lack of EV molecular markers specific to tumor cells; - no longitudinal analysis |
| Osti D et al,  Clin Cancer Res, 2019  [10] | n=43 GB patients;  n=9 GB patients with relapse;  n=25 patients with other CNS malignancies | n=33 | EDTA | Pre-OP;  3 days post-OP | Differential  centrifugation | NTA (LM10/532-nm laser)  TEM  WB  Proteomic analysis | No difference | - quantitative, but not qualitative assessment of plasma EVs; - no longitudinal analysis |
| Döring K et al,  In Vivo,  2024  [30] | n=36 GB patients | no | EDTA | Pre-OP;  Post-OP;  stable disease; progressive disease | Ultracentrifugation | NTA (ZetaView PMX-120/640 nm laser) | Not indicated | - small cohort; - no HCs; - no longitudinal analysis |
| Present study | n=21 GB patients | n=8 | Sodium  citrate | Pre-OP;  Post-OP;  Follow-up 3, 6, 9 months post-OP | Differential  centrifugation | NTA (NS300/488 nm laser)  WB | GB > HCs | - small cohort; |

Pre-OP: preoperative, Post-OP: postoperative, CNS: Central Nervous System, EVs: Extracellular Vesicles, GB: Glioblastoma, HCs: healthy controls, IFCM: Imaging Flow Citometry, NTA: Nanoparticle Tracking Analysis, TEM: Transmission Electron Microscopy, WB: Western Blotting.


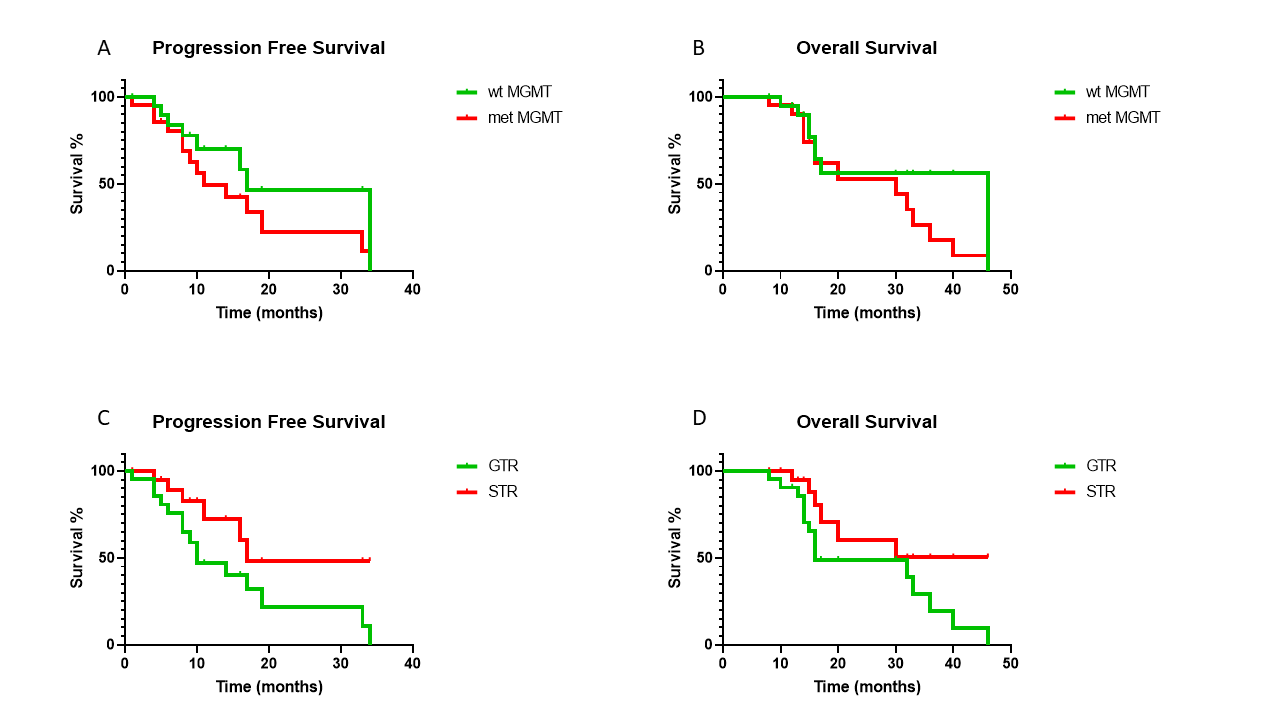


**Figure S1:** Kaplan-Meier survival curves. Survival in months of patients selected, expressed as a percentage, based on MGMT promoter status non hypermethylated (wt MGMT) or hypermethylated (met MGMT) in Progression Free Survival (A) or Overall Survival (B). Survival in months of patients selected, expressed as a percentage, based on Gross Total Resection (GTR) or Subtotal Resection (STR) in Progression Free Survival (C) or Overall Survival (D).


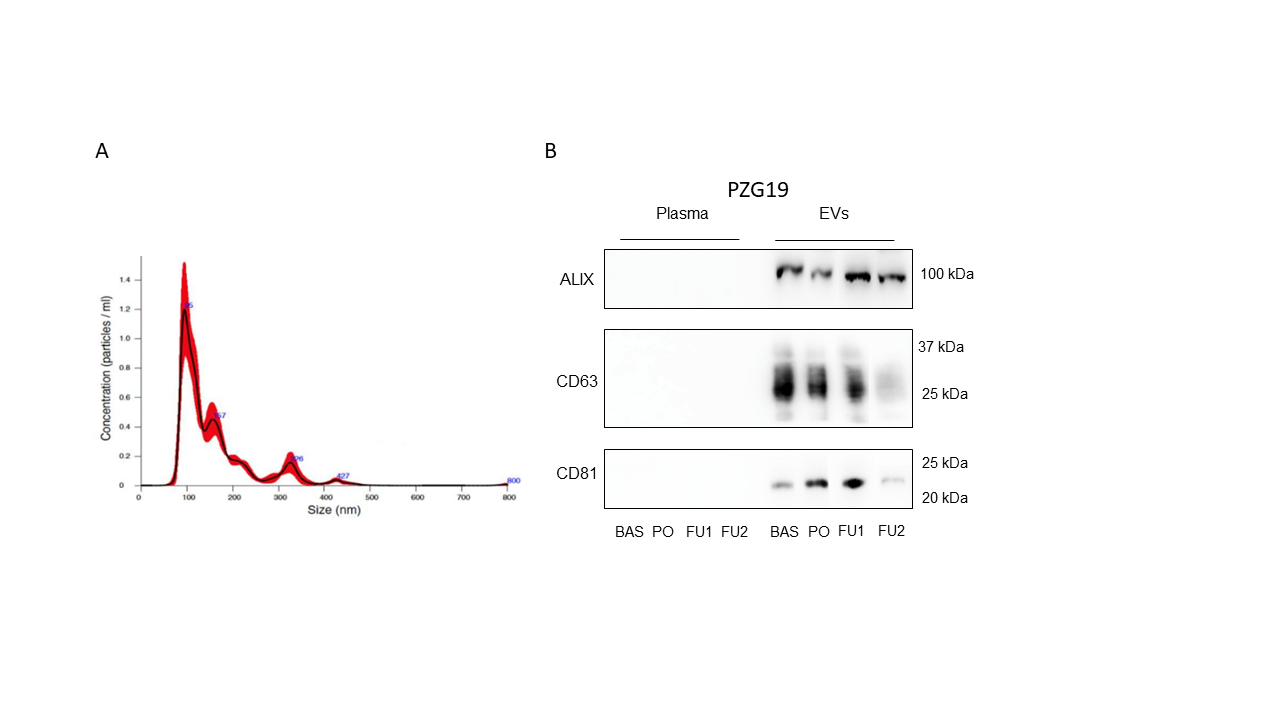


**Figure S2:** Extracellular vesicles characterization. A) Nanoparticle Tracking Analysis (NTA) of plasma EVs. A representative sample is shown. Concentration (particle/mL) and size distribution (nm) of EVs is analyzed. B) Enrichment of EVs markers: WB analysis of ALIX, CD63 and CD81 in EVs at different timepoints (BAS: preoperative, PO: postoperative, FU1: follow up 1, FU2: follow up 2 respect to plasma sample from which they derived. A representative sample is shown.

**
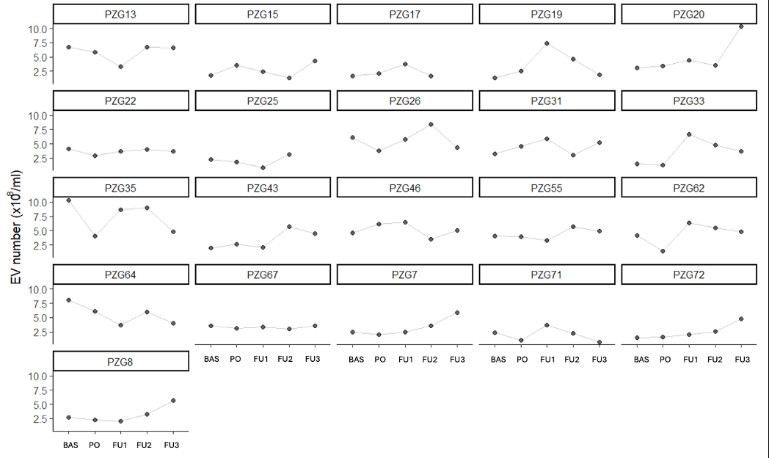
**

**Figure S3: Longitudinal trend of plasma EV concentration for all patient enrolled**. The lines show the EV concentration (particle/plasma ml) for each patient enrolled in the study. The black dots represent the time of plasma collection, Basal: a day before the surgical resection, PO, postoperative: 2-3 days after surgery, FU1 (Follow up1): 3 months after surgery, FU2 (Follow up2): 6 months after surgery, FU3 (Follow up3): 9 months after surgery. “PZG” indicates patient.
